# Systematic mapping of global research on climate and health: a machine learning review

**DOI:** 10.1016/S2542-5196(21)00179-0

**Published:** 2021-07-14

**Authors:** Lea Berrang-Ford, Anne J Sietsma, Max Callaghan, Jan C Minx, Pauline F D Scheelbeek, Neal R Haddaway, Andy Haines, Alan D Dangour

**Affiliations:** a Priestley International Centre for Climate, University of Leeds, Leeds, UK; b Mercator Research Institute on Global Commons and Climate Change, Berlin, Germany; c Centre on Climate Change and Planetary Health, London School of Hygiene & Tropical Medicine, UK; d Stockholm Environment Institute, Stockholm, Sweden; e Africa Centre for Evidence, University of Johannesburg, Johannesburg, South Africa

## Abstract

**Background:**

The global literature on the links between climate change and human health is large, increasing exponentially, and it is no longer feasible to collate and synthesise using traditional systematic evidence mapping approaches. We aimed to use machine learning methods to systematically synthesise an evidence base on climate change and human health.

**Methods:**

We used supervised machine learning and other natural language processing methods (topic modelling and geoparsing) to systematically identify and map the scientific literature on climate change and health published between Jan 1, 2013, and April 9, 2020. Only literature indexed in English were included. We searched Web of Science Core Collection, Scopus, and PubMed using title, abstract, and keywords only. We searched for papers including both a health component and an explicit mention of either climate change, climate variability, or climate change-relevant weather phenomena. We classified relevant publications according to the fields of climate research, climate drivers, health impact, date, and geography. We used supervised and unsupervised machine learning to identify and classify relevant articles in the field of climate and health, with outputs including evidence heat maps, geographical maps, and narrative synthesis of trends in climate health-related publications. We included empirical literature of any study design that reported on health pathways associated with climate impacts, mitigation, or adaptation.

**Findings:**

We predict that there are 15 963 studies in the field of climate and health published between 2013 and 2019. Climate health literature is dominated by impact studies, with mitigation and adaptation responses and their co-benefits and co-risks remaining niche topics. Air quality and heat stress are the most frequently studied exposures, with all-cause mortality and infectious disease incidence being the most frequently studied health outcomes. Seasonality, extreme weather events, heat, and weather variability are the most frequently studied climate-related hazards. We found major gaps in evidence on climate health research for mental health, undernutrition, and maternal and child health. Geographically, the evidence base is dominated by studies from high-income countries and China, with scant evidence from low-income counties, which often suffer most from the health consequences of climate change.

**Interpretation:**

Our findings show the importance and feasibility of using automated machine learning to comprehensively map the science on climate change and human health in the age of big literature. These can provide key inputs into global climate and health assessments. The scant evidence on climate change response options is concerning and could significantly hamper the design of evidence-based pathways to reduce the effects on health of climate change. In the post-2015 Paris Agreement era of climate solutions, we believe much more attention should be given to climate adaptation and mitigation options and their effects on human health.

**Funding:**

Foreign, Commonwealth & Development Office.

## Introduction

The effects of climate change on health are already evident in populations worldwide and threaten to undermine the past 50 years of global gains in public health.^1^ Health can be affected by the changing climate through many causal pathways, including the direct effects of heat and extreme weather events,^2^, ^3^, ^4^ ecosystem-mediated effects (including through food systems^5^, ^6^, ^7^, ^8^ and the distribution of vectors that transmit diseases such as dengue or malaria^9^, ^10^) and effects mediated by socioeconomic pathways, such as increased poverty, population displacement, and conflict.^11^, ^12^ Climate change is also expected to weaken coping strategies (eg, mental health capacity, livelihood diversification, use of emergency assets, water conservation, and crop rotation), especially among poor communities in low-income countries.^3^, ^13^, ^14^

Identification of the most crucial links between climate change and health to enable mitigation and adaptation responses that support health is underpinned by timely and regularly updated review of the scientific evidence base.^15^, ^16^, ^17^ Two factors constrain the availability of such evidence reviews. First, research on climate and health takes place across various disciplines and silos, representing a fragmented landscape of niche discourses that hinders efforts to synthesise key insights and identify trends and evidence gaps. Second, exponentially increasing literature means that conventional evidence synthesis methods that typically require considerable human resources to manually collate and screen literature are no longer sufficient or feasible.^18^, ^19^, ^20^ Indeed, faced with this dilemma, many evidence syntheses have responded by narrowing their review focus, reviewing an increasingly smaller portion of the literature, and further compromising the potential for broader insights across disciplinary silos.^1^, ^20^, ^21^

**Research in context**

**Evidence before this study**

Substantial efforts to track and assess the links at the global level between climate and health are underway. An example is the Intergovernmental Panel on Climate Change (IPCC)'s assessment reports, which synthesise and update scientific research every 5–8 years on the effects of climate on health, and adaptation and mitigation responses and synergies related to health. The IPCC reports attempt to synthesise the state of evidence, but can only include a small fraction of the literature base, which is large and increasing exponentially. To date, there is no comprehensive evidence using machine-learning methods for systematic evidence mapping or synthesis to capture the breadth of the literature on this topic.

To contextualise this study and identify previous similar work, we searched for publications that used systematic methods to synthesise the global literature on climate and health. We used a wide definition of systematic methods, including systematic reviews, meta-analyses, meta-syntheses, scoping or mapping reviews, bibliometric analyses, and other approaches with explicit and transparent review methodologies. For peer-reviewed articles published in English between Jan 1, 2013, and April 9, 2020, we did a rapid title and abstract review of literature indexed in Web of Science and Scopus for articles including the topic terms “climat*” AND “health*” AND “systematic” (all years), and in PubMed using the MeSH terms “climate change” AND “systematic review” (all years). We screened for articles that were global in scope and not restricted to particular health outcomes, climate-related drivers, or particular populations. We found studies reviewing the relationship between climate and health (using a range of systematic approaches) for specific regions and nations, climate-related hazards, and health outcomes or determinants.

Our search returned only one relevant published article without geographical or topic restrictions that provided a bibliometric analysis of global climate and health literature between 2003 and 2018. Bibliometric results focused largely on authorship networks, additionally identifying frequent keywords and keyword co-occurrence. Their results showed scarce published studies outside of high-income nations and China, and substantial overlap of keywords across articles. The bibliometric analysis used a single database (Web of Science) and a narrow set of search terms (retrieving 6719 articles). It used bibliometric analysis methods that did not include screening or coding of articles, and did not apply machine learning approaches. There is also an emerging literature drawing on the synthesis of national or urban policy documents to empirically assess adaptation or mitigation policy, or both, related to the effects of climate change on health.

**Added value of this study**

This is the first study to combine machine learning and systematic evidence mapping approaches to comprehensively describe and map the global scientific literature on climate and health. Given the exponential increase in literature on climate and health, almost all conventional systematic mapping approaches have narrowed their geographical or topical scope to maintain the robustness of systematic methods. The literature on automation in evidence synthesis has mainly focused on specific machine learning interventions in different stages of the systematic review process. We leverage machine learning to more accurately identify and classify relevant literature at both the screening and analysis stages, reflecting significant advancements on standard bibliometric methods, and showing the potential benefit of these methods to support multiple stages of the assessment pipeline.

We build on our previous work, and show how evidence mapping can be scaled to vast literatures that characterise entire research fields through the development of a (semi)automated machine learning pipeline. Such automation is crucial for assessments of the scientific literature on climate and health, where individuals and groups of authors can no longer manually manage increasing volumes of literature, and where conventional systematic mapping approaches increasingly introduce bias through increasingly restrictive inclusion criteria.

**Implications of all the available evidence**

The integration of machine learning with systematic evidence mapping approaches can help to maintain transparency and scientific scrutiny of scientific assessments as we move into an era of big literature. Our comprehensive map of the literature on climate and health, and the methods we used, can contribute to ongoing initiatives to assess the full breadth of the scientific evidence base in a robust and systematic way, including the IPCC, the *Lancet* Countdown, or the Pathfinder Commission. Our results additionally provide a basis for identifying and highlighting key research and knowledge gaps and prioritising allocation of research funding and resources. National and international institutions are investing in climate change mitigation and adaptation, with climate funding expected to increase substantially over the next decade. To ensure effective and adequate preparedness and mitigation of the health impacts of climate change, governments urgently need a robust evidence base to guide, prioritise, and justify interventions. Our synthesis shows geographical variation in evidence about health effects, with vulnerable regions under-represented, and key gaps in evidence across a number of climate-health pathways.

In an age of big literature, new approaches are needed to systematically synthesise the available evidence in a timely manner.^1^, ^18^, ^22^, ^23^, ^24^ Machine learning techniques can rapidly screen and code potentially hundreds of thousands of articles, enabling the breadth and diversity of the expanding literature base to be considered.^1^, ^25^, ^26^, ^27^

We aimed to use machine learning methods (supervised and unsupervised) to systematically synthesise the evidence base on climate change and human health, including the impacts of climate change on health, climate change mitigation, and adaptation responses relevant to health. In doing so, we hope to provide the first comprehensive, semi-automated systematic map of the scientific literature on climate change and human health.

## Methods

### Search strategy and selection criteria

We used machine learning approaches to systematically synthesise evidence on the associations between human health and climate change, climate variability, and weather (CCVW), globally. The protocol for this analysis has been published online.^28^ Briefly, our analysis involved five steps: defining the framework, database searching, screening and supervised machine learning, unsupervised machine learning, and generating topic maps and heatmaps.

We defined key concepts using the Intergovernmental Panel on Climate Change definitions of risk, impacts, hazards, exposure, vulnerability, and adaptation and mitigation responses. We focused on global literature, but we assessed the distribution of literature based on country income status, using the 2020 World Bank income classification rankings to define low-income, lower middle-income, upper middle-income, and high-income nations.

We included all empirical study designs published and indexed in English between Jan 1, 2013, and April 9, 2020, reflecting publishing since the Intergovernmental Panel on Climate Change's fifth assessment report. We searched Web of Science Core Collection, Scopus, and PubMed using title, abstract, and keywords only. Full texts were not retrieved, screened, or analysed. Grey literature were not included. A detailed description of search strings for Scopus is in appendix 1 (pp 1–3). All searches were done using the NACSOS platform.

### Screening

Screening was done based on titles and abstracts using a combination of manual assessment against a set of a priori inclusion criteria and machine learning methods. For inclusion, documents had to meet the following criteria: be indexed in English; be published between Jan 1, 2013, and April 9, 2020; provide a clear link to actual, projected, or perceived impacts of climate change, responses to reduce the impacts of climate change (adaptation), or the mitigation of greenhouse gas emissions; include substantial focus on a perceived, experienced, or observed eligible health-related outcome or health system; and present empirically driven research or a review (including non-systematic reviews) of such research. Detailed screening criteria and a ROSES checklist for systematic mapping are available online.

We used supervised machine learning to facilitate screening. This approach is based on the concept that a computer algorithm can be trained to predict the decisions that would be made by a human screener or coder. Team members manually screened a training sample of abstracts (3730 unique documents of which 2100 were randomly sampled), and coded them as relevant to the impacts, adaptation, or mitigation categories, or any combination of the three. 11% (n=410) of the training sample was reviewed by multiple team members, and differences were discussed until a consensus was reached. This human-screened sample set was used to train a supervised machine learning algorithm to predict for the remaining articles (1) whether each article is likely to be relevant to our inclusion and exclusion criteria and (2) how the document should be categorised (impacts, adaptation, or mitigation). We compared several supervised learning algorithms from the Scikit-learn package^29^ (appendix 1 pp 4–6), calculating the performance of the algorithm (accuracy, precision, and recall) using 10 k-fold cross-validation. We found that a support vector machine provided the highest accuracy, with a relatively even distribution between precision and recall. We also used a support vector machine in a one-versus-rest set-up to make the category predictions.

### Data extraction and analysis

We extracted the bibliographic meta-data for all documents retrieved through search strings. To determine where studies were taking place, we used a pre-trained geoparser^30^ to identify geographical locations in the abstract and title. Bibliographic data for all included literature are available online.

We used a method of unsupervised machine learning, topic modelling, to support analysis of literature included in this review. Topic modelling is a method that automatically identifies clusters of words that frequently occur together based on a pre-specified number of topics.^31^ The themes resulting from topic modelling are not based on any human labelling or tagging, they are instead based on structures that the algorithm finds in the data itself. An article is typically associated with multiple topics. To find the most relevant and interpretable topic model, we qualitatively compared several alternative topic models, using the Scikit Learn implementation of latent dirichlet allocation^32^ and non-negative matrix factorisation,^33^ with 40–80 topics and various hyperparameters (appendix 1 p 7). Non-negative matrix factorisation with 70 topics provided the best balance between detail and interpretability. We iteratively reviewed the topics emerging from the topic models to identify and label key thematic clusters that were then validated within the study team and with external experts to ensure that final topics were parsimonious with expert understanding of the literature. The topics of the final topic model were assigned to one of five aggregated meta-topics: climate hazards (ie, CCVW), health risks and impacts, options and responses, mediating pathways, or other (typically methods topics).

We generated topic maps based on the outcomes of the topic model and created heat maps to visualise the relative co-occurrence of topics. We identified the most frequent and least frequent topics from among all topics in our topic model by global region for key meta-topics. We generated geographical maps to visualise the locations of studies globally. We used narrative synthesis to assess the frequency of key topics within the climate and health literature, as well as the extent of co-occurrence of topics within the topic and heat maps. We assessed the extent to which trends in the literature differ by country income class. Source code for both machine learning and data analyses are available online.

### Role of the funding source

The funder of the study reviewed and provided comments on the study protocol and draft results. The funder of the study had no role in data collection, data analysis, data interpretation, or writing of the report.

## Results

**Figure 1:**
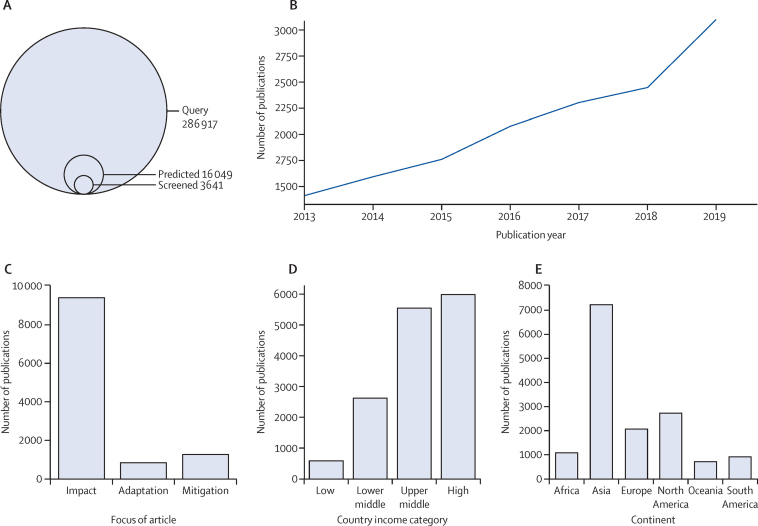
Descriptive summary of included articles (A) Sampling frame, indicating the number of articles that were manually screened by investigators and those that were predicted to be relevant (final dataset for inclusion), compared with the initial number of documents retrieved from search string queries. (B) For relevant abstracts, trends in publications over time indicate a continued increase in the volume of literature on climate and health. Literature published between Jan 1, 2013, and April 9, 2020, were included. Bar graphs show the number of publications by impact, adaptation, and mitigation categories (C), national income category as per World Bank classifications (D), and global regions (E).

Our findings showed that the literature on climate and health is vast and growing quickly (figure 1). Based on our inclusion criteria, we predict that there are 15 963 studies in the field of climate and health published between 2013 and 2019 and indexed in Web of Science Core Collections, Scopus, or PubMed. Between Jan 1 and Dec 31, 2019, 3128 relevant studies were retrieved and included—twice as many as were included only 6 years before in 2013—with the literature growing on average by 14% per year.^18^, ^34^ This dataset can be found in appendix 2.

Climate-health literature is dominated by impact studies, with only a minority of studies focusing on solutions and co-benefits (figure 1). 13 380 (84%) of 15 963 available studies scored highest on the topic of impacts of CCVW on (or associations with) human health. Studies focusing on the health impacts of human responses were a minority: 1660 (10%) studies on mitigation and 1189 (7%) studies on adaptation. The most frequently reported co-benefits were ancillary benefits to human health from climate change mitigation. We found that few studies focused substantively on the benefits to climate change mitigation or adaptation in the health sector.

12 629 (79%) of 15 914 studies on climate and health with identified place names focus on high-income and upper middle-income nations, particularly China (figure 1). Publishing on climate change and health shows a strong gradient by national income group. The number of publications from high-income nations was two-times greater than the number from lower middle-income nations, and close to ten-times the number from low-income nations. The number of publications from upper middle-income nations was similar to those from high-income countries, a finding that is largely driven by a high number of publications from China. When place mentions are combined by country for each paper, we still find that 1704 (17%) of the remaining 9739 locations are in China, and 1382 (14%) documents include at least one location in the USA.

**Figure 2:**
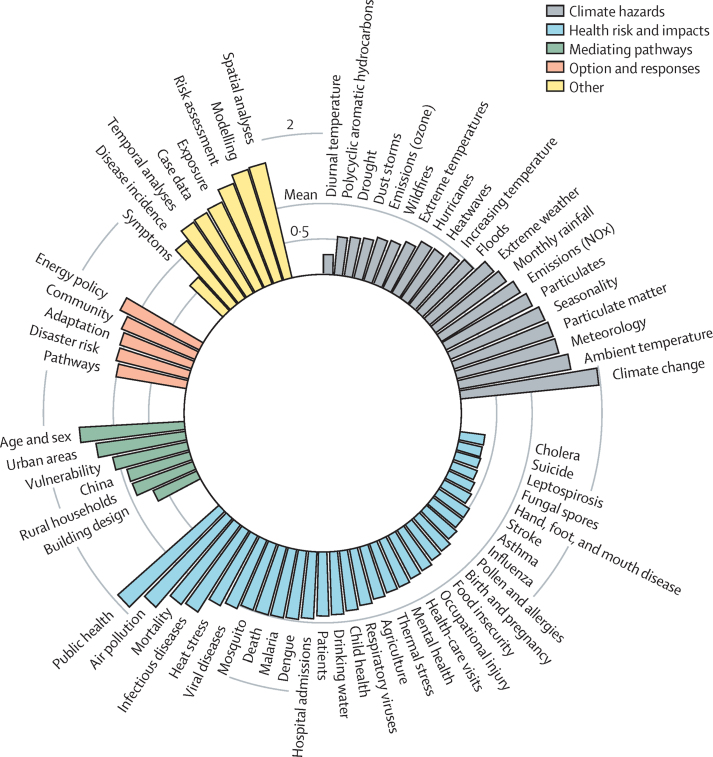
Prevalence of topics within included articles, organised by meta-topic The axis is a normalised scale that reflects topic prevalence relative to the mean score (reference=1). For example, a bar with a value on the axis of 2 would mean that topic is twice as prevalent as the mean of all topics. A bar with a value of 0·5 would be a topic that occurs half as often as the mean. Topics are identified based on words used in article titles, keywords, and abstracts, and can thus reflect several meanings. Community, for example, includes articles related to community resilience, community perceptions, community-level studies, and community participation. Viewing the detailed words within this topic (appendix 1 p 7) shows that much of the literature driving this topic is associated with community and resilience as dominant co-occurring words.

**Figure 3:**
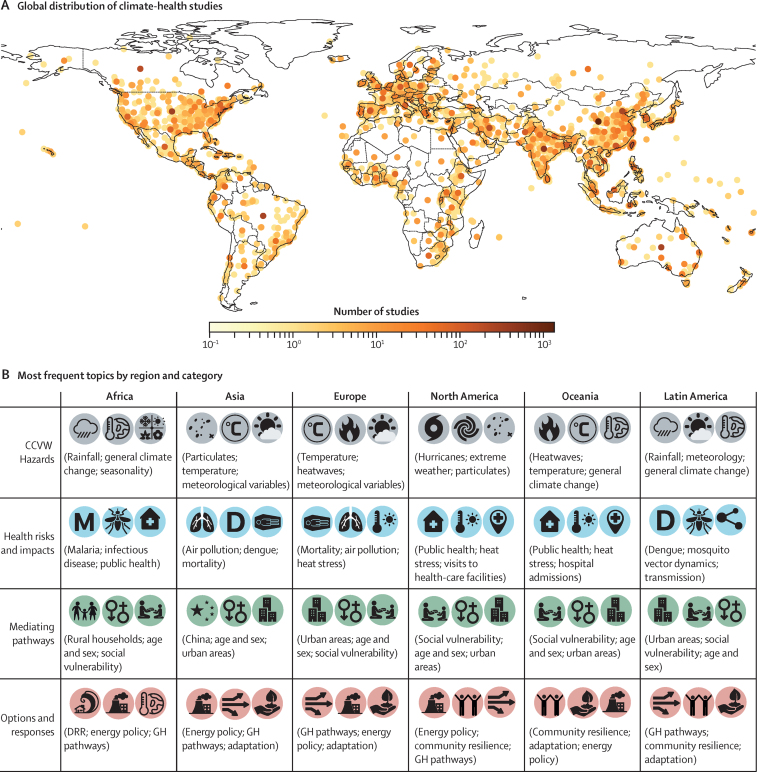
Geographical distribution of included studies where location information was available (A) and most frequent topics by region and category (B) Legend in map shows total number of articles. For studies conducted at the national level, points appear in the geographic centre of the region or country. CCVW=climate change, climate variability, and weather. DRR=disaster risk reduction. GH=greenhouse.

Using topic modelling, we mapped key hazards, health impacts, mediating pathways and risk modifiers, and responses across global regions. The resulting 70 topics, with their relative prevalence in the literature, are shown in figure 2, grouped by hazards, impacts, mediating pathways and risk modifiers, responses, and other. Full lists of topics and words associated with these topics are in the appendix 1 (p 7). The other category was dominated by topics (and associated words) related to methods, reflecting a strong focus on quantitative statistical analysis in the literature. Some topics, such as public health or climate change, reflect the generic use of terms such as health, climate, and impact across the literature. The topics with the highest frequency by region (top three topics) are shown in figure 3.

Hazards topics include meteorological variability, the role of extreme events, and particulate emissions (Figure 2, Figure 3). As a proxy, we calculated the number of documents that at least partially focus on any given topic, although these numbers are only meaningful in a relative sense (ie, ie, to estimate the size of the literature on a topic relative to the size of that on another topic). Most reported hazards related to extreme events—including 1022 (6%) of all 15 963 articles included language on floods, hurricanes (793 [5%]), heatwaves (831 [5%]), drought (564 [4%]), dust storms (554 [3%]), and wildfires (613 [4%])—or air quality conditions, including particulate matter (1986 [12%]), NOx and vehicle emissions (1658 [10%]), ozone emissions (690 [4%]), and polycyclic aromatic hydrocarbons (394 [2%]). Other reported hazards include meteorological variation—such as rainfall (1888 [12%] of 15 963 articles) and extreme or increasing temperatures (1364 [9%])—and seasonality (2318 [15%]). The effect of particulates on air quality was a particularly prevalent topic in Asia and Europe. Extreme events were among the top three hazard topics in North America, where hurricanes were the most prevalent topic. Heatwaves were major hazard topics in Europe and Oceania. Literature published in Africa and Latin America more frequently reported on rainfall and meteorological variability than other CCVW hazards, compared with other regions.

We found that health topics are dominated by air quality, all-cause mortality, infectious disease, and heat stress (Figure 2, Figure 3). Various different health outcomes are reported, with a substantial focus on respiratory effects—including air pollution (2548 [16%] of 15 963 articles), respiratory viruses (1035 [6%]), asthma (459 [3%]), pollen and allergies (265 [2%])—heat stress (1410 [9%]) and infectious diseases, particularly vector-borne infectious diseases including dengue (867 [5%]), malaria (722 [5%]), influenza (345 [2%]), cholera (172 [1%]), and leptospirosis (162 [1%]). Respiratory health was the most frequent health topic in Asia, and heat stress was one of the top three most frequent topics in Europe, North America, and Oceania. A large portion of the literature is focused on CCVW predictors of all-cause mortality, particularly in Asia and Europe. Malaria, dengue, influenza, leptospirosis, and cholera are all frequent disease-specific topics, with malaria being the top health topic in Africa, and dengue being the top health topic in Latin America and the second most frequent topic in Asia. The most frequent health topics in Latin America and Africa are infectious diseases (particularly vector-borne infectious diseases). Public health (3089 [19%] of all 15 963 articles) was a highly prevalent topic in several regions, with a frequent focus on increased pressure on health-care facilities. Water and sanitation (1097 [7%]), agriculture and food insecurity (839 [5%]), mental health (730 [5%]), maternal and child health (1149 [7%]), and occupational health and injury (728 [5%]) also emerged as common health topics, but were not the leading topics in any region.

Mediating pathways include social vulnerability and urban exposure to heat risk and infectious disease (Figure 2, Figure 3). Mediating pathways and risk modifiers for human health risks from CCVW are dominated by the topic of age and sex (occurring in 2442 [15%] of all 15 963 articles), and were among the top three topics in all regions. An emergent topic focused on health in urban areas (1710 [11%]), particularly heat risk due to urban heat islands in Europe and urban infectious disease risk in Latin America. Socially mediated vulnerability (1406 [9%]) emerged as a frequent topic in all regions except Asia, and was the top mediating pathway in literature on North America and Oceania. The only geography-based topic to emerge among top topics was a specific focus on China, with much of this literature exploring associations between emissions, air quality, and respiratory health. Rural households (1151 [7%]) and building design (706 [4%]) were also among mediating pathway topics, particularly in the context of stove use and air quality. Rural households was the most frequent pathway topic in Africa.

Within the available mitigation and adaptation literature, energy policy (1669 [10%] of all 15 963 articles) was the most common topic and, together with greenhouse gas emission pathways, was among the top three response topics in all regions except Oceania and Latin America (Figure 2, Figure 3). Literature on mitigation topics also included pathways to emissions reductions and future climate scenarios (1183 [7%]). Adaptation topics focused on disaster risk reduction (1248 [8%]), community resilience (1541 [10%]), and adaptation policy and practice (1366 [9%]). Disaster risk reduction was the top response topic in Africa. Community resilience and adaptation policy and practice were top topics in Oceania and Latin America.

**Figure 4:**
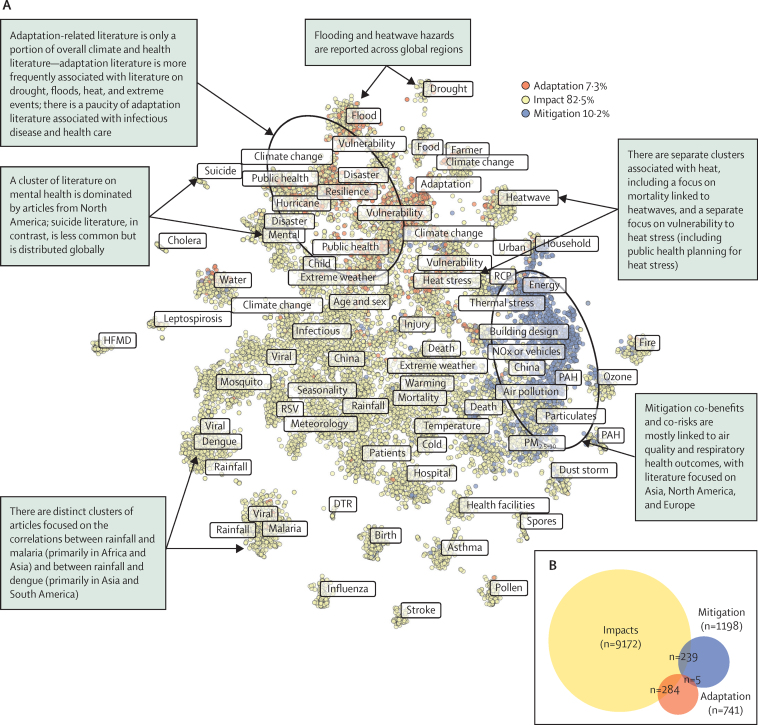
Visualisation of topics and climate research categories in the dataset (A) Topic map in which each dot represents a document, coloured according to the categories of impact, adaptation, or mitigation. There are no axes per se; the graphic reflects a conceptual space where similar documents are placed closer together, and dissimilar documents are farther apart. Clusters of dots represent areas of literature that have similar topic scores, meaning that they use similar words and are presumed to be about related subjects. Labels show the most frequent topics. Arrow boxes show illustrative trends emerging from the map. (B) Summary of the number of documents in each category, and the number of documents that span multiple categories. Numbers are based on machine learning predictions (ie, assigned a score of >0·5 by the classifier). DTR=diurnal temperature range. HFMD=hand, foot, and mouth disease. PAH=polycyclic aromatic hydrocarbons. PM=particulate matter. RCP=representative concentration pathways. RSV=respiratory syncytial virus. In the case of adaptation and to some extent mitigation, these are likely underestimates. Up to 36% of adaptation abstracts and 18% of mitigation abstracts might be misclassified as impacts articles, based on 10 k-fold cross-validation. Even when accounting for this, only a minority of articles focus on adaptation or mitigation compared with impacts, and only five articles focus on both mitigation and adaptation.

We saw poor integration of research on impacts, mitigation, and adaptation across key topics (figure 4). We mapped all articles in our dataset based on the similarity of topics within documents—ie, distinguishing documents based on their primary category: impacts, mitigation, or adaptation. The topic map reflects a conceptual space where similar documents are placed closer together and dissimilar documents are farther apart; thus clusters of dots represent areas of literature that have similar topic scores. The topic map shows a large number of clusters of impact-related topics, including several focused on specific health outcomes (eg, malaria, influenza, suicide, and stroke) that are highly clustered and separate from other topics. CCVW-related topics, such as seasonality, meteorology, and temperature, show less distinctive clustering. Of the health topics, heat stress and air quality appear to be the most strongly integrated with CCVW-related topics. Mitigation topics are fewer and clustered together, with substantial overlap between mitigation and air pollution topics. Adaptation clusters are relatively uncommon and under-represented. Notably, there was no substantive overlap of adaptation topics with mitigation topics, indicating negligible attention to co-benefits and co-risks across these two dominant response options. The health areas most strongly clustered with adaptation topics include food and mental health, with many health topics showing negligible proximity to adaptation clusters. Some topics occur twice or more within figure 4, reflecting their association with different and separate areas of literature. For example, the topic of vulnerability occurs in three areas of distinct literature: once in proximity to heat stress and Representative Concentration Pathways (denoting different climate change scenarios), once close to climate change, adaptation, and resilience, and once close to floods and disasters.

**Figure 5:**
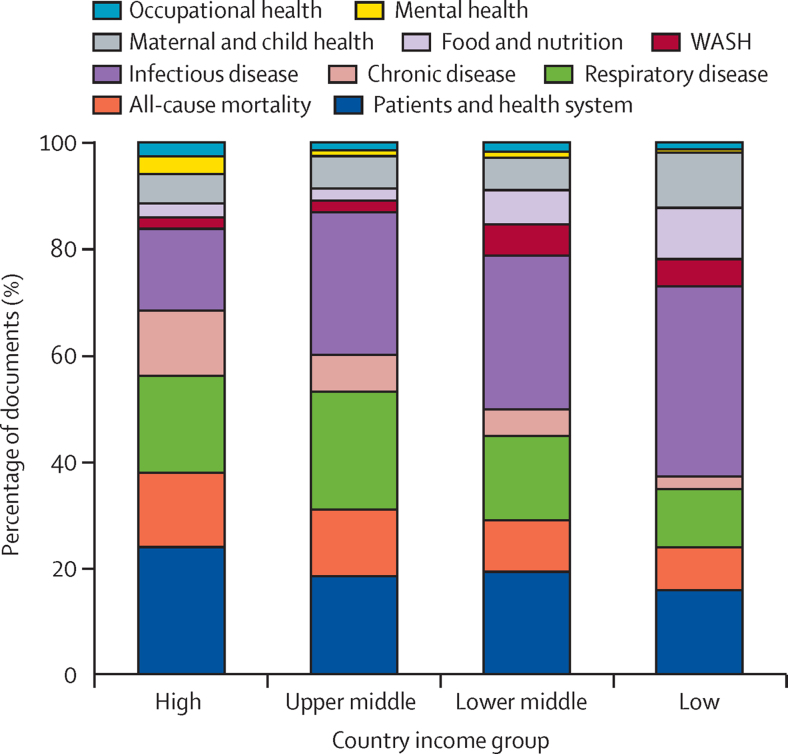
Frequency of health risk and impact topics for countries in different income classes Data are from documents on health impacts per country income group, subdivided by aggregated topic as a percentage of the group total. WASH=water, sanitation, and hygiene.

With higher national income level, there is a gradient of decreasing focus on infectious disease, maternal and child health, and nutritional illness, and an increasing focus on chronic disease, health system demand, and respiratory health (figure 5). Compared with low-income and middle-income regions, literature from high-income regions had a greater focus on the effects of CCVW on hospital admissions, chronic disease, and pressures on the health system, including all-cause mortality due to heat, poor air quality, and extreme events. In low-income regions, infectious diseases dominate the literature on climate and health, with strong additional foci on food and nutrition and maternal and child health. There is a clear gradient of increasing emphasis on infectious disease; food and nutrition; water, sanitation, and hygiene; and maternal and child health with lower income status; this relationship is paralleled by a gradient of decreasing emphasis on chronic disease, respiratory health, and health systems demand. One notable exception to this gradient is a greater focus on respiratory health in upper middle-income countries, particularly driven by literature from China.

**Figure 6:**
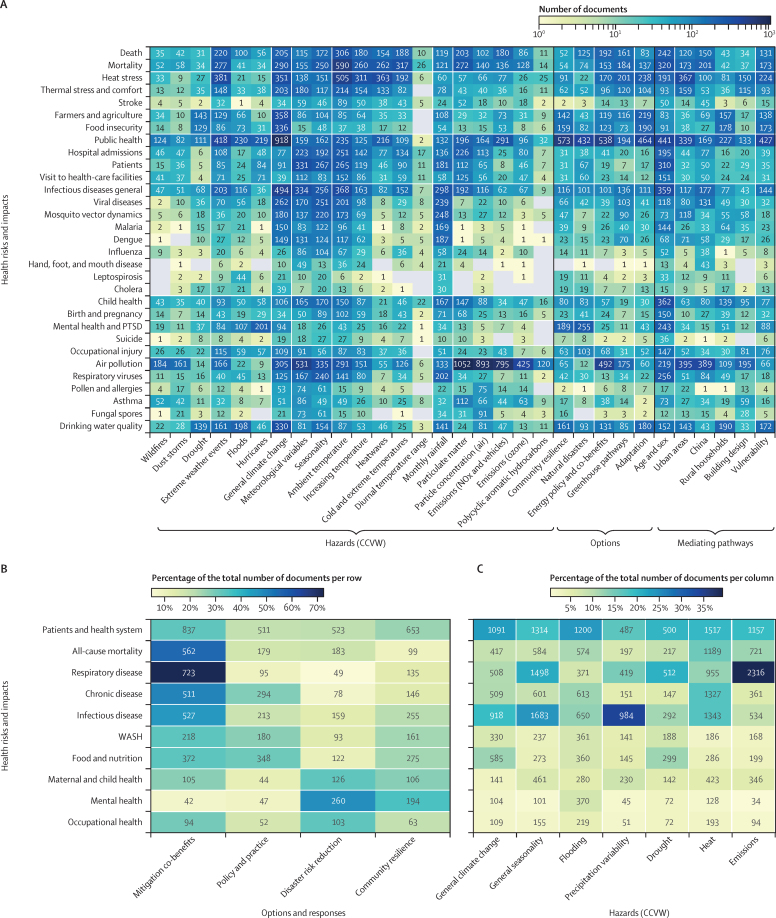
Heat maps showing the co-occurrence of documents by individual topics (A) and aggregated categories (B, C) (A) Detailed co-occurrence of topics by health risks and impacts versus hazards, options and responses, and mediating pathways. Aggregated health categories versus options and responses (B) and aggregated hazard categories (C). All heat maps give the number of documents classified by the topic model as including both topics within the same document. A document is counted when the topic score is above 0·015. Colour scale set by the percentage of the total number of documents per row for (B) and by the column total for (C). CCVW=climate change, climate variability, and weather. PTSD=post-traumatic stress disorder. WASH=water, sanitation, and hygiene.

We assessed the co-occurrence of topics by hazard, health, mediating pathway, and response option categories, with results shown as heat maps (figure 6A–C). Co-occurrence of hazard and health topics was common, which largely reflects dominant causal pathways for climate-health risk. For example, precipitation variability and heat were frequently reported topics in articles on infectious diseases, reflecting an emphasis on research on rainfall correlates of vectorial capacity for vector-borne diseases, such as malaria and dengue. Similarly, emissions topics frequently co-occurred with studies of respiratory health, reflecting the association between air quality and respiratory health risks. Chronic disease topics and topics related to health-care system demand and all-cause mortality most frequently co-occurred with heat hazard topics. Among more specific topics (figure 6A), air pollution co-occurred frequently with meteorological variability, particularly temperature. Infectious diseases were more broadly associated with variation in temperature and rainfall, and extreme weather events. Wildfires and dust storms often co-occurred with literature on respiratory health, including air pollution, and asthma, particularly among children. Drought literature most frequently co-occurs with impacts on water quality, food insecurity, and agriculture, which probably reflects the role of decreased rainfall in increasing water contamination and reducing agricultural productivity. Heatwaves and heat were frequently reported in articles on all-cause mortality, heat stress, thermal comfort, and health systems demand. Mental health literature frequently co-occurred with a focus on extreme events, including hurricanes and floods.

Among mediating pathways and risk factors, age and sex frequently co-occurred with child health outcomes, and maternal and child health, reflecting the unique risks associated with women and children (figure 6A). Studies in urban areas co-occur strongly with air pollution and heat, whereas studies of rural households more frequently focus on food, agriculture, child health, and water quality. Literature focused on China is dominated by studies of air pollution, all-cause mortality, and infectious disease. Studies exploring building design most frequently co-occur with heat and air pollution, reflecting attention to the role of infrastructure design in supporting thermal comfort and air quality in indoors settings. Studies linked to social vulnerability showed a similar co-occurrence profile to rural households, with literature focusing on food, agriculture, and water quality. Social vulnerability also frequently co-occurred with studies of heat stress and infectious disease.

Options and responses to the human health impacts of climate change included strong co-occurrence of literature on the respiratory health co-benefits of mitigation (figure 6B). Co-benefits of mitigation also co-occurred strongly with all-cause mortality, chronic disease, and infectious diseases. Although energy policy more frequently co-occurred with air pollution and short-term health benefits, greenhouse gas pathways co-occurred more strongly with longer-term health effects, such as heat stress and infectious disease (figure 6B). Among health topics, mental health co-occurred most strongly with adaptation topics, particularly disaster risk reduction and community resilience, reflecting research on the role of disasters and disaster recovery in affecting mental health outcomes. Similarly, maternal and child health and occupational health frequently co-occurred with disaster risk reduction responses, reflecting the potential effects of extreme events on injury and the unique risks to women and children. Adaptation policy and practice co-occurred most frequently with studies of health-care system demand, reflecting strategic health systems planning. Chronic diseases; water, sanitation, and hygiene; and food and nutritional health topics also co-occurred frequently with adaptation policy and practice and, to a lesser extent, community resilience.

## Discussion

Our findings reveal novel geographically distributed patterns in the published evidence base linking climate change and health. We identified a predominance of evidence from high-income and upper middle-income countries, and a markedly diverse prevalence of topics across world regions, reflecting national burdens of disease and regional engagement with, and capacity to conduct, relevant research. The contrasting patterns of evidence across different country income groups highlights the need for localised responses to the effects of climate change on health, and the large evidence base emerging from China shows the changing nature of research priorities with increasing national incomes.

We noted multiple evidence gaps, most notably under-representation of evidence from central Asia, north and central Africa, and South America. The disconnect in foci of research is also a concern. In Africa, for example, research is largely focused on vector-borne disease and public health systems, despite the potential for substantial climate change impacts on maternal and child health, respiratory infections, and nutritional deficiencies—which are the first, second, and 11th causes, respectively, of disability-adjusted life-years lost in Africa in 2019.^35^ The relative absence of evidence on mental health, including the effects of agricultural shifts and extreme events on livelihood instability, and the implications of environmental migration on cultural and social cohesion are also a concern. The social determinants of climate impacts on health, and modifiable entry points for intervention, are under-represented among the top topics in the literature. Further, the paucity of evidence on both climate change mitigation and adaptation (alone and in combination) is of great concern, and unless urgently resolved will limit the ability of governments to design evidence-based pathways to reduce the effects on health of climate change.

Our approach to systematic evidence mapping rooted in machine learning provides a much more comprehensive and specific view of the evidence on the climate and health nexus than existing work,^36^ and as such provides a new response to the big literature challenge.^1^, ^18^, ^24^ However, limitations in data availability and accessibility require important trade-offs and potentially create biases that could have been avoided by the strictest level of systematic review and mapping standards. We did not include grey literature, which remains difficult to integrate systematically using machine learning methods. We used only three bibliographic databases (Web of Science, Scopus, and PubMed), and relied for all analyses on abstracts, titles, and keywords only. Finally, we restricted our search to studies indexed in English. The resulting evidence map, for example, follows a common pattern with little evidence in Africa and South America that has been observed in evidence mapping efforts on other topics in similar ways,^18^, ^20^ and might partially relate to a language bias. Similarly, the over-representation of evidence on high-income and middle-income countries could be partially related to the focus on peer-reviewed publications.

In summary, reliance on the meta-data of studies highlights the importance of adequate reporting by authors to enable research discovery using machine learning methods. The development of curated climate-health vocabularies (eg, MeSH), structured abstracts, and author awareness of how meta-data affect research discovery will be important in further enabling the potential of machine learning for evidence synthesis. New approaches to timely evidence synthesis will be necessary if researchers and policy makers are to keep up with rapid transitions in climate policy and a growing evidence base. The integration of methodological standards in systematic review with new machine learning approaches will be key to advancing evidence synthesis over the next decade. Development and curation of living evidence platforms, for example, are feasible and represent potentially cost-effective opportunities to support decision-making to prepare for, and reduce the current and future effects of, climate change on health.

## Data sharing

The NACSOS platform and source code for this work are available online. Extended materials are available at https://doi.org/10.5281/zenodo.4352030. Data in this study take the form of published journal articles. All study data (ie, journal article citations) are available at https://doi.org/10.5281/zenodo.4972515. Requests for further access to study data should be made to the corresponding author.

## Declaration of interests

We declare no competing interests.

## References

[1] Minx JC, Callaghan M, Lamb WF, Garard J, Edenhofer O (2017). Learning about climate change solutions in the IPCC and beyond. Environ Sci Policy.

[2] Sheridan SC, Allen MJ (2018). Temporal trends in human vulnerability to excessive heat. Environ Res Lett.

[3] Gautier D, Denis D, Locatelli B (2016). Impacts of drought and responses of rural populations in West Africa: a systematic review. Wiley Interdiscip Rev Clim Change.

[4] Jolly WM, Cochrane MA, Freeborn PH (2015). Climate-induced variations in global wildfire danger from 1979 to 2013. Nat Commun.

[5] Phalkey RK, Aranda-Jan C, Marx S, Höfle B, Sauerborn R (2015). Systematic review of current efforts to quantify the impacts of climate change on undernutrition. Proc Natl Acad Sci USA.

[6] Scheelbeek PFD, Bird FA, Tuomisto HL (2018). Effect of environmental changes on vegetable and legume yields and nutritional quality. Proc Natl Acad Sci USA.

[7] Alae-Carew C, Nicoleau S, Bird FA (2020). The impact of environmental changes on the yield and nutritional quality of fruits, nuts and seeds: a systematic review. Environ Res Lett.

[8] Zhao C, Liu B, Piao S (2017). Temperature increase reduces global yields of major crops in four independent estimates. Proc Natl Acad Sci USA.

[9] Xu L, Stige LC, Chan KS (2017). Climate variation drives dengue dynamics. Proc Natl Acad Sci USA.

[10] Beck-Johnson LM, Nelson WA, Paaijmans KP, Read AF, Thomas MB, Bjørnstad ON (2017). The importance of temperature fluctuations in understanding mosquito population dynamics and malaria risk. R Soc Open Sci.

[11] Haines A, Ebi K (2019). The Imperative for Climate Action to Protect Health. N Engl J Med.

[12] Intergovernmental Panel on Climate Change (2018). Global warming of 1.5°C. An IPCC special report on the impacts of global warming of 1.5 °C above pre-industrial levels and related global greenhouse gas emission pathways, in the context of strengthening the global response to the threat of climate change, sustainable development, and efforts to eradicate poverty. https://www.ipcc.ch/site/assets/uploads/sites/2/2019/06/SR15_Full_Report_High_Res.pdf.

[13] Mycoo MA (2018). Beyond 1.5 degrees C: vulnerabilities and adaptation strategies for Caribbean Small Island Developing States. Reg Environ Change.

[14] Bhatta GD, Aggarwal PK (2016). Coping with weather adversity and adaptation to climatic variability: a cross-country study of smallholder farmers in South Asia. Clim Dev.

[15] Araos M, Austin SE, Berrang-Ford L, Ford JD (2016). Public health adaptation to climate change in large cities: a global baseline. Int J Health Serv.

[16] Ebi KL, Boyer C, Bowen KJ, Frumkin H, Hess J (2018). Monitoring and evaluation indicators for climate change-related health impacts, Risks, Adaptation, and Resilience. Int J Environ Res Public Health.

[17] Lesnikowski AC, Ford JD, Berrang-Ford L, Paterson JA, Barrera M, Heymann SJ (2011). Adapting to health impacts of climate change: a study of UNFCCC Annex I parties. Environ Res Lett.

[18] Callaghan MW, Minx JC, Forster PM (2020). A topography of climate change research. Nat Clim Chang.

[19] Haddaway NR, Callaghan MW, Collins AM (2020). On the use of computer-assistance to facilitate systematic mapping. Campbell Syst Rev.

[20] Lamb WF, Creutzig F, Callaghan MW, Minx JC (2019). Learning about urban climate solutions from case studies. Nat Clim Chang.

[21] Lamb WF, Callaghan MW, Creutzigt F, Khosla R, Minx JC (2018). The literature landscape on 1.5 degrees C climate change and cities. Curr Opin Environ Sustain.

[22] Berrang-Ford L, Döbbe F, Garside R (2020). Editorial: evidence synthesis for accelerated learning on climate solutions. Campbell Syst Rev.

[23] Berrang-Ford L, Pearce T, Ford JD (2015). Systematic review approaches for climate change adaptation research. Reg Environ Change.

[24] Nunez-Mir GC, Iannone BV, Pijanowski BC, Kong N, Fei S (2016). Automated content analysis: addressing the big literature challenge in ecology and evolution. Methods Ecol Evol.

[25] Sietsma AJ, Ford JD, Callaghan MW, Minx JC (2021). Progress in climate change adaptation research. Environ Res Lett.

[26] Cheng SH, Augustin C, Bethel A (2018). Using machine learning to advance synthesis and use of conservation and environmental evidence. Conserv Biol.

[27] Nakagawa S, Samarasinghe G, Haddaway NR (2019). Research weaving: visualizing the future of research synthesis. Trends Ecol Evol.

[28] Berrang-Ford L, Sietsma AJ, Callaghan M (2021). Mapping global research on climate and health using machine learning (a systematic evidence map). Wellcome Open Res.

[29] Pedregosa F, Varoquaux G, Gramfort A (2011). Scikit-learn: machine Learning in Python. J Mach Learn Res.

[30] Halterman A (2017). Mordecai: full text geoparsing and event geocoding. J Open Source Softw.

[31] Lesnikowski A, Belfer E, Rodman E (2019). Frontiers in data analytics for adaptation research: Topic modeling. Wiley Interdiscip Rev Clim Change.

[32] Blei DM, Ng AY, Jordan MI (2003). Latent dirichlet allocation. J Mach Learn Res.

[33] Cichocki A, Phan A-H (2009). Fast local algorithms for large scale nonnegative matrix and tensor factorizations. IECE T Fund Electr.

[34] Grieneisen ML, Zhang M (2011). Correspondence: the current status of climate change research. Nat Clim Chang.

[35] Institute for Health Metrics and Evaluation (2015). GBD Compare at Viz Hub. http://vizhub.healthdata.org/gbd-compare.

[36] Watts N, Amann M, Arnell N (2021). The 2020 report of the *Lancet* Countdown on health and climate change: responding to converging crises. Lancet.

