# Supplementary material for: Systematic mapping of global research on climate and health: a machine learning review
Source: Lancet Planet Health. 2021 Jul 14;5(8):e514–25. doi: 10.1016/S2542-5196(21)00179-0 (PMC8358978; doi:10.1016/S2542-5196(21)00179-0)
Supplement: Supplementary appendix 1 [file mmc1.pdf]

THE LANCET
Planetary Health

Supplementary appendix

This appendix formed part of the original submission and has been peer reviewed. We post it as supplied by the authors.

Supplement to: Berrang-Ford L, Sietsma AJ, Callaghan M, et al. Systematic mapping of global research on climate and health: a machine learning review. *Lancet Planet Health* 2021; published online July 13. https://doi.org/10.1016/ S2542-5196(21)00179-0.

APPENDIX 1 – SUPPLEMENTARY MATERIALS

Table of Contents

Summary of search strings …………………………………………………………………………………………………… 1

Extended materials for machine learning methods ………………………………………………………………… 4

List of topics from the topic model, and associated categorisations, as well as keywords dominant within each topic ……………………………………………………………………………………………………. 7

APPENDIX I: SUPPLEMENTARY MATERIALS

Summary of search strings.

*Each of the strings is connected by a boolean 'OR'. The Scopus search string is given here; for Web of Science and Medline, the syntax is different, and some other minor changes were made, most notably removing left-truncated keywords. Search hits shown in the table were conducted on 9 April 2020. Note the following data search functions: * = any subsequent letters; W/# = maximum number of words allowed between the term directly to the left and that directly to the right of the W/#; and ? = any letter or space to replace the "?".*

Theme	Key concepts	String (Scopus)	Attributable Hits (scopus)
**Climate change**<br>*(contains at least one of the following climate terms, from any category)*	General climate change terms	(climat* OR "global warming" OR "greenhouse effect*")	35,052
	Greenhouse gasses, including short-lived greenhouse gasses, and particulate matter when linked to emission or mitigation. Some astronomy results are excludedfiltered out.	(("carbon dioxide" OR co2 OR methane OR ch4 OR "nitrous oxide" OR n2o OR "nitric oxide" OR "nitrogen dioxide" OR nox OR *chlorofluorocarbon* OR *cfc* OR refrigerant OR hydrofluorocarbon* OR hfc* OR *chlorocarbon* OR "carbon tetrachloride" OR ccl4 OR halogen* OR ozone OR o3 OR ammonia OR nh3 OR "carbon monoxide" OR co OR "volatile organic compounds" OR nmvoc OR "hydroxyl radical" OR "oh" OR "pm2.5" OR aerosol OR "black carbon" OR "organic carbon" OR "sulphur dioxide" OR "oxidized sulphur" OR "so2" OR "sox" OR "sulphuric acid" OR so4*) W/2 (emit* OR emission OR releas* OR mitigat*) AND NOT(star OR "solar system"))	7,871
	Climate variability indicators/climate indices	(temperature* OR precipitat* OR rainfall OR "heat ind*" OR "extreme-heat event*" OR "heat-wave" OR "extreme-cold*" OR "cold ind*" OR humidity OR drought* OR hydroclim* OR monsoon OR "el ni$o" OR enso OR SOI OR "sea surface temperature*" OR sst)	199,558

	Complex climate indices, including extreme weather events, floods, wildfire, and coastal changes. Some paleo-climatic events are excluded.	(snowmelt* OR flood* OR storm* OR cyclone* OR hurricane* OR typhoon* OR "sea-level" OR wildfire* OR "wild-fire*" OR "forest-fire*" OR ((extreme W/1 event*) AND NOT paleo*) OR "coast* erosion" OR "coastal change*" OR (disaster* W/1 (risk OR manag* OR natural)))	22,031
**AND**<br><br>**Health**<br><br>*(contains at least one of the following health terms, from any category)*	General health terms	(health* OR well?being OR ill OR illness OR disease* OR syndrome* OR infect* OR medical*)	49,773
	General health outcomes	(mortality OR daly OR morbidity OR injur* OR death* OR hospital* OR {a&e} OR emergency OR emergencies OR doctor OR gp)	33,571
	Nutrition, including obesity and undernutrition	(obes* OR over?weight OR under?weight OR hunger OR stunting OR wasting OR undernourish* OR undernutrition OR anthropometr* OR malnutrition OR malnour* OR anemia OR anaemia OR "micronutrient*" OR "micro?nutrient*" OR diabet*)	2,239
	Cardio-vascular terms. Some studies on Chemical Vapour Deposition (CVD) are excluded.	(hypertension OR "blood pressure" OR stroke OR *vascular OR (cvd AND NOT(vapour or vapor)) OR "heart disease" OR isch?emic OR cardio?vascular OR "heart attack*" OR coronary OR chd)	6,047
	Renal health terms	(ckd OR renal OR cancer OR kidney OR lithogenes*)	4,934

	Effects of temperature extremes	((heat W/2 (stress OR fatigue OR burn* OR stroke OR exhaustion OR cramp*)) OR skin OR fever* OR renal* OR rash* OR eczema* OR "thermal stress" OR hypertherm* OR hypotherm*)	23,846
	Maternal health outcomes	(pre?term OR stillbirth OR birth?weight OR lbw OR maternal OR pregnan* OR gestation* OR *eclampsia OR sepsis OR oligohydramnios OR placenta* OR haemorrhage OR hemorrhage)	2,041
	Vector-borne diseases	(malaria OR dengue* OR mosquito* OR chikungunya OR leishmaniasis OR encephalit* OR vector-borne OR pathogen OR zoonos* OR zika OR "west nile" OR onchocerciasis OR filariasis OR lyme OR tick?borne)	2,257
	Bacterial, parasitic and viral infections, including waterborne and foodborne diseases	(waterborne OR “water borne” OR diarrhoea* OR diarrhe*l OR gastro* OR enteric OR *bacteria* OR viral OR *virus* OR parasit* OR vibrio* OR cholera OR protozoa* OR salmonella OR giardia OR shigella OR campylobacter OR food?borne OR aflatoxin OR poison* OR ciguatera OR((snake* OR adder*) W/2 bite*))	46,064
	Respiratory outcomes	(respiratory OR allerg* OR lung* OR asthma* OR bronchi* OR pulmonary* OR copd OR rhinitis OR wheez*)	3,432
	Mental health outcomes	(mental OR depress* OR *stress* OR anxi* OR ptsd OR psycho* OR *trauma* OR suicide* OR solastalgi*)	12,616
	Health systems	[no additional terms needed]	

Extended materials for machine learning methods

1 Supervised learning

Supervised learning was used to create a dataset of relevant documents. The final code for this can be found at https://doi.org/10.5281/zenodo.4322697. In short, we tried four different machine learning algorithms, all through implementations of the Scikit-learn toolkit. These were a Neural Net, Naïve Bayes, Random Forrest and Support Vector Machine (SVM). We chose initial hyper-parameters based on the advised starting value of the Scikit documentation and prior experience with similar task. We then used 10 k-fold cross validation to arrive at the scores of the figure below.

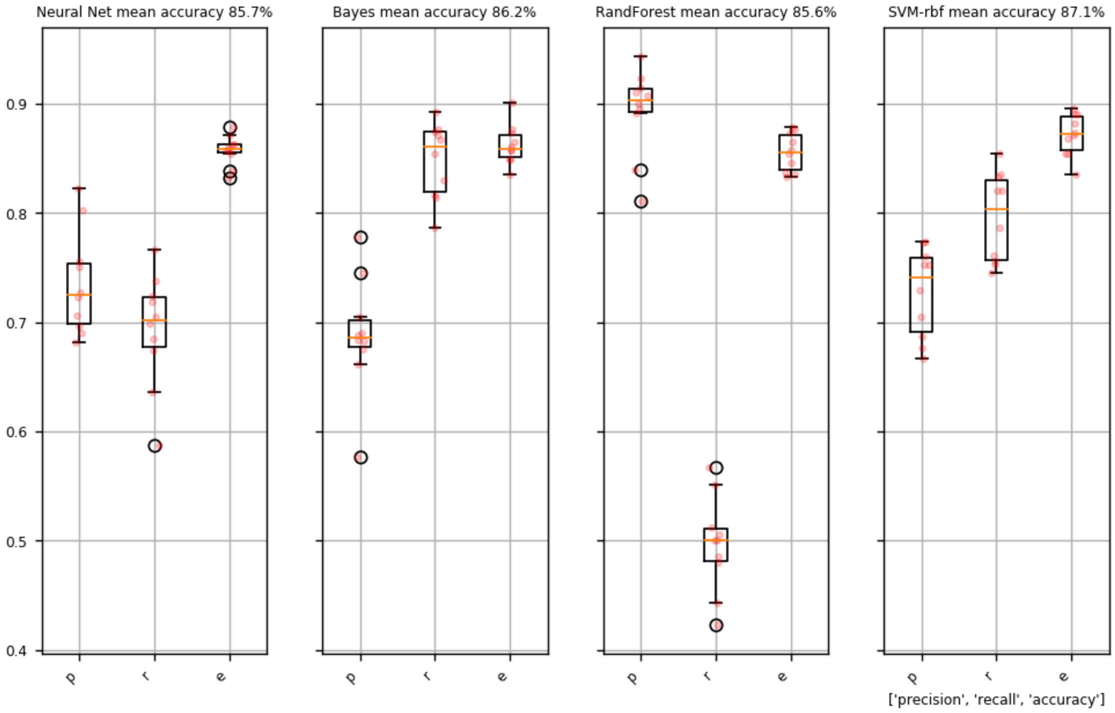

As can be seen, SVM gives promising results here, with the highest accuracy and a good spread of precision and recall, without any real outliers. Based on this, we ran a few extra test runs with different kernels (linear and RBF) as well as class weights (balanced and unbalanced). The RBF kernel and balanced weights proved to give the best results. More extensive parameter tuning was not done at this stage. SVM was also used to predict classes in a one-vs-rest set-up.

Since we used the algorithm to provide relevance scores (rather than a Boolean in/out), we could then investigate what threshold for inclusion would be appropriate. Since our aim was to be fairly comprehensive, it seemed prudent to lower the threshold for inclusion somewhat, gaining recall at

the expense of some precision. From the precision/recall plot (below), this appeared a plausible strategy.

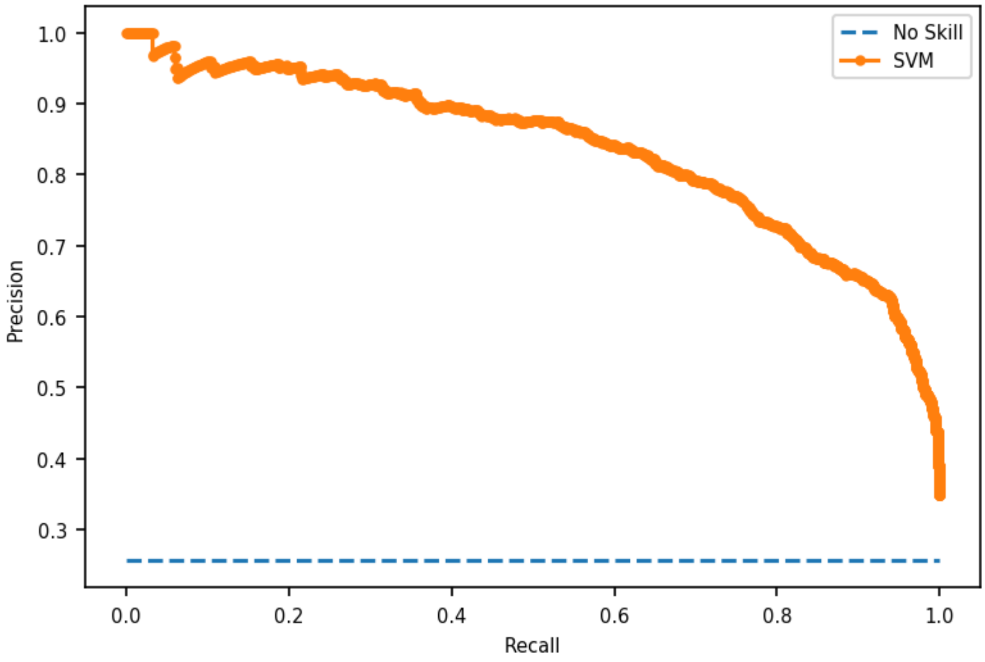

By lowering the threshold to 0.35, mean precision was still at 70%, while recall rose to 84%. Crucially, when we ran a topic model on this group of documents and calculated which topics gained most documents by this lower threshold, we found that this affected especially adaptation and vulnerability-related topics, which seemed crucial to include, given the projects' goals. The document set with the lower inclusion threshold was therefore chosen as the final dataset.

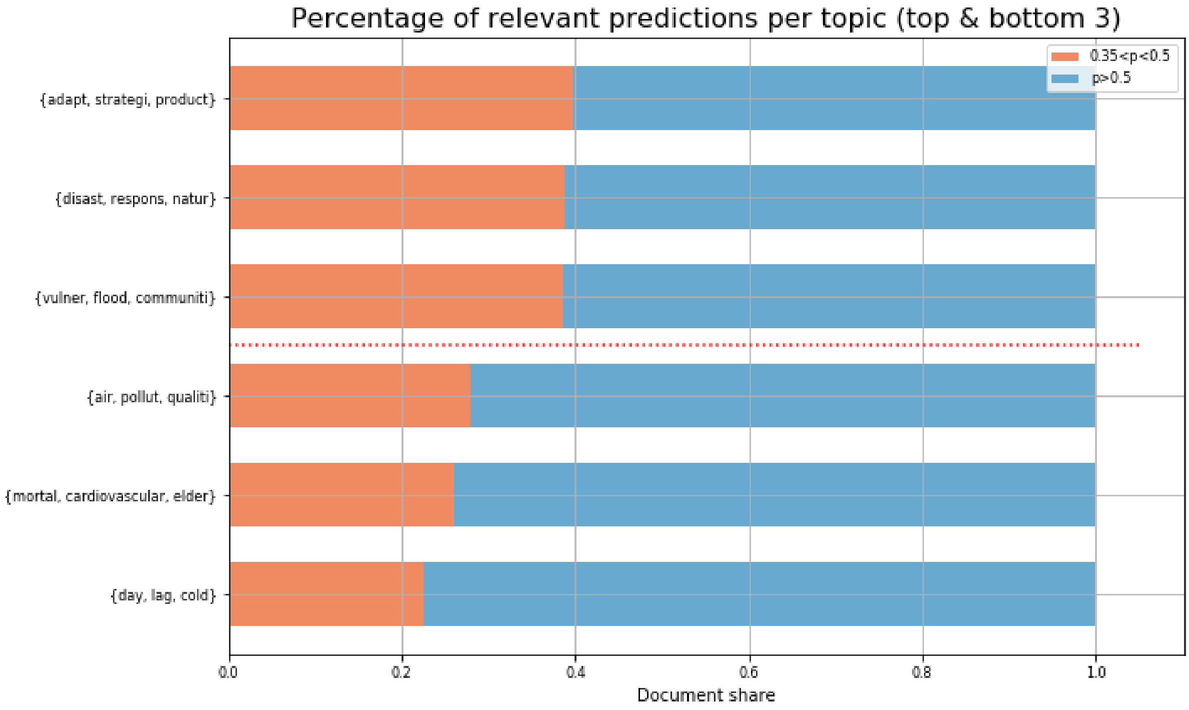

2 Unsupervised learning

For topic modelling, we ran two different algorithms with a variety of parameters: Latent Dirichlet Allocation (LDA) and Non-negative Matrix Factorization (NMF). For both, we use implementations provided by Scikit-learn. In an initial run, we tried both algorithms for 40-80 topics in intervals of 5 topics. For NMF, the alpha parameter (regularization term) was set at 0.01, while for LDA, alpha (concentration parameter of Dirichlet distribution, also called theta) was set at $k/50$, where k is the number of topics. The latter is taken from (Griffiths and Steyvers, 2004).

Investigation of the resulting topic models showed that NMF was consistently better at separating health topics from climate topics – LDA appeared to struggle to find health topics especially. We therefore ran additional NMFs with increasing alpha (0.01, 0.05, 0.1, 0.5 and 1). Results appeared to be relatively robust to changes in this parameter, but an alpha of 0.1 provided a slightly cleaner separation of topics.

Moreover, the ideal number of topics appeared to likely be at the higher end of our initial range. We therefore compared an NMF with alpha set to 0.1 for 60-80 topics, again at intervals of 5 topics. 70 topics provided best balance between detail and interpretability and was therefore chosen as the final model.

GRIFFITHS, T. L. & STEYVERS, M. 2004. Finding scientific topics. *Proceedings of the National Academy of Sciences of the United States of America,* 101 Suppl 1**,** 5228-5235.

List of topics from the topic model, and associated categorisations, as well as keywords dominant within each topic. Topic prevalence is a ratio reflecting how often each topic occurs relative to the average topic (>1 more prevalent than average; <1 less prevalent).

Aggregated meta-topic	Aggregated topic	Topic name	Top 3 stemmed keywords	Topic prevalence
Climate hazards	Drought	Wildfires	fire, wildfir, smoke	0.96
		Dust storms	dust, storm, desert	0.85
		Drought	drought, impact, veget	0.81
	Extreme precipitation & flooding	Extreme weather events	extrem, event, weather	1.71
		Floods	flood, damag, river	1.43
		Hurricanes	hurrican, evacu, sandi	1.21
	General climate change	General climate change	climat, chang, impact	2.86
	General seasonality & weather	Meteorological variables	meteorolog, humid, correl	2.16
		Seasonality	season, winter, summer	2.09
	Heat	Ambient temperature	temperatur, effect, ambient	2.33
		Increasing temperatures	degre, increas, temperatur	1.35
		Heatwaves	heatwav, definit, dure	1.22
		Cold & extreme temperatures	cold, spell, hot	1.07
		Diurnal temperature range	dtr, diurnal, effect	0.40
	Precipitation variabilty	Monthly rainfall	month, rainfal, period	1.80
	Emissions	Particulate matter	particulatematt, particul, matter	2.14
		Particulate concentration (air	concentr, particl, site	1.99
		Emissions (Nox & vehicles)	emiss, nox, vehicl	1.90
		Emissions (ozone)	ozon, concentr, ppb	0.92
		PAH	pah, sourc, combust	0.78
Health risks & impacts	All-cause mortality	Death	death, caus, excess	1.45
		Mortality	mortal, cardiovascular, allcaus	2.05
	Chronic	Heat stress	heat, stress, heatrel	1.65
		Thermal stress & comfort	thermal, stress, comfort	1.09
		Stroke	stroke, ischem, hemorrhag	0.62
	Food & nutrition	Farmers & agriculture	farmer, crop, agricultur	1.13
		Food insecurity	food, insecur, secur	0.93
	Patients & health systems	Public health	health, public, review	2.79
		Hospital admissions	hospit, admiss, respiratori	1.40
		Patients	patient, acut, medic	1.34
		Visits to health care facilities	visit, emerg, outpati	1.02
	Infectious	Infectious diseases general	diseas, infecti, respiratori	1.98
		Viral diseases	transmiss, outbreak, virus	1.54
		Mosquito vector dynamics	mosquito, vector, abund	1.45
		Malaria	malaria, transmiss, falciparum	1.44
		Dengue	dengu, fever, outbreak	1.43
		Influenza	influenza, epidem, week	0.76
		HFMD	hfmd, foot, mouth	0.58
		Leptospirosis	leptospirosi, leptospira, human	0.52
		Cholera	cholera, outbreak, epidem	0.51
	Maternal & child health	Child health	children, child, age	1.27
		Birth & pregnancy	birth, preterm, pregnanc	0.91
	Mental health	Mental health & PTSD	mental, ptsd, disord	1.07
		Suicide	suicid, associ, rate	0.51
	Occupational health & injury	Occupational injury	injuri, worker, occup	1.00
	Respiratory	Air pollution	pollut, air, qualiti	2.42
		Respiratory viruses	infect, rsv, respiratori	1.20
		Pollen & allergies	pollen, allergen, airborn	0.82
		Asthma	asthma, childhood, exacerb	0.75
		Fungal spores	spore, fungal, airborn	0.54
	WASH	Drinking water quality	water, drink, sourc	1.32
Options & responses	Developing community resilience	Community resilience	communiti, resili, social	1.63
	Disaster risk reduction	Disaster risk reduction	disast, natur, prepared	1.53
	Mitigation co-benefits	Energy policy & co-benefits	energi, cost, polici	1.82
		Greenhouse pathways	scenario, project, rcp	1.46
	Policy & practice	Adaptation	adapt, strategi, plan	1.55
Mediating pathways	Demographic vulnerability	Age & Sex	age, preval, group	2.17
	Geographic exposure	Urban areas	urban, citi, uhi	1.86
		China	china, provinc, haze	1.35
		Rural households	household, stove, rural	1.33
		Building design	indoor, outdoor, home	0.99
	Social vulnerability	Vulnerability	vulner, social, hazard	1.57
Other	Methods & mixed	Spatial analyses	spatial, area, region	2.40
		Modeling & forecasting	model, predict, forecast	2.37
		Risk assessment & risk perc	risk, factor, percept	2.20
		Exposure	exposur, associ, estim	1.94
		Case data	case, report, number	1.89
		Temporal analyses	day, lag, associ	1.87
		Disease incidence	incid, rate, provinc	1.65
		Symptoms	symptom, depress, associ	0.92
